# ^1^H-NMR-based metabolomics reveals that prior exercise modulates metabolic changes in the cerebral cortex and hippocampus in sleep-deprived mice

**DOI:** 10.1590/1414-431X2025e14816

**Published:** 2026-03-30

**Authors:** B.R.D. da Silva, P.I.G. Nunes, R.S. de Matos, L.M.A. Silva, E.G. Alves, E.S. de Brito, P.F.C. de Bruin, V.M.S. de Bruin

**Affiliations:** 1 Programa de Pós-Graduação em Ciências Médicas, Faculdade de Medicina, Universidade Federal do Ceará, Fortaleza, CE, Brasil; 2 Empresa Brasileira de Pesquisa Agropecuária, Fortaleza, CE, Brasil; 3 Departamento de Engenharia de Alimentos, Centro de Ciências, Universidade Federal do Ceará, Fortaleza, CE, Brasil; 4 Empresa Brasileira de Pesquisa Agropecuária, Maceió, AL, Brasil

**Keywords:** Brain, Exercise, Metabolomics, NMR, Sleep deprivation

## Abstract

Sleep deprivation induces profound metabolic disturbances in the brain, while regular physical exercise is recognized for promoting neuroprotection and energy balance. This study investigated the effects of chronic treadmill exercise on the cortical and hippocampal metabolic profiles of sleep-deprived Swiss mice using the entire ^1^H-NMR spectrum. Male mice (n=48) were assigned to four groups: Control, Exercise (EX), Sleep Deprivation (SD), and Exercise before Sleep Deprivation (EX+SD). The EX group underwent 8 weeks of aerobic training, and SD was induced by 72-h total sleep deprivation. Brain metabolomic analysis revealed that the cortex and hippocampus shared a qualitatively similar metabolic composition, with taurine, creatine, and lactic acid as the most abundant metabolites. Exercise increased cortical levels of lactic acid, creatine, taurine, and other metabolites involved in glycolysis, the TCA cycle, and osmoregulation, while SD disrupted energy-related metabolites and increased glial markers such as myo-inositol. The EX+SD group exhibited a cortical metabolic profile similar to controls, indicating that prior exercise preserved neuroenergetic balance in this region. In contrast, hippocampal metabolism remained partially affected by SD, despite exercise preconditioning. These findings suggest that exercise confers region-specific metabolic resilience, especially in the cortex, by modulating pathways related to pyruvate metabolism, glutamate turnover, and astrocytic-neuronal coupling. Regular physical activity may thus act as a non-pharmacological strategy to mitigate SD-induced neurochemical imbalances.

## Introduction

Sleep deprivation (SD), a prevalent condition in modern society, is frequently associated with impairments such as attention deficits, anxiety, depression, and cognitive decline. Chronic sleep loss also contributes to the development of serious medical conditions, including obesity, diabetes, cardiovascular diseases, dementia, and obstructive sleep apnea (1,2). Certain occupational groups, such as physicians, nurses, firefighters, and air traffic controllers, are particularly vulnerable to SD due to extended working hours and shift work (1,3). It is estimated that approximately one-third of adults do not obtain sufficient sleep (2,4). Therefore, SD affects a large portion of the world population, with many patients remaining undiagnosed and untreated (1,2). Biological factors like aging and comorbidities may exacerbate SD-related consequences, while environmental factors, such as noise, stress, ambient light, and electronic device exposure, can further aggravate sleep disturbances (1,2).

Regular physical exercise improves sleep quality and plays a critical role in mitigating pathological conditions by enhancing sleep architecture and cognitive performance. Recent studies have demonstrated the beneficial impact of exercise on several neurological disorders, including Parkinson's disease and bipolar disorder (5). Furthermore, a meta-analysis confirmed that physical activity enhances behavioral performance and promotes neurogenesis, both in healthy individuals and in those with dementia, possibly reducing the toxicity and formation of cerebral amyloid plaques (6). O'Brien et al. (7), using plasma metabolomic and lipidomic profiling, reported that intense physical training attenuates the deleterious effects of SD. Nevertheless, current evidence regarding exercise-induced modulation in brain inflammation, oxidative stress, and metabolism under sleep-deprived conditions remains limited (8).

Previous studies suggest that SD alters the expression of genes and proteins involved in cellular stress responses, energy metabolism, neurotransmission, and synaptic plasticity in rodent models (9). However, transcriptomic and proteomic analyses face limitations in capturing post-translational modifications and in assessing specific protein subtypes. In contrast, metabolomics provides a more integrative and functional snapshot of the biological system, reflecting the real-time cellular activity and offering a comprehensive view of the organismal phenotype (10).

Metabolomics involves the comparative analysis of endogenous metabolites in biological samples under defined physiological or pathological conditions. This technique has gained wide application in biomedical research due to its high sensitivity, capacity to detect subtle metabolic changes, and potential to uncover novel biomarkers and underlying metabolic pathways (11).

We focused our analysis on the cortex and hippocampus, two brain regions critically involved in memory consolidation, executive function, and emotional regulation, processes well-documented as vulnerable to SD (12). These regions also exhibit high basal metabolic activity and demonstrate substantial metabolic alterations following sleep loss, rendering them suitable for analysis via hydrogen nuclear magnetic resonance (^1^H-NMR) spectroscopy (13).

Despite existing challenges in standardizing animal models and experimental designs, metabolomics represents a promising strategy to elucidate the metabolic impact of SD and physical exercise in both preclinical and clinical contexts (14). These insights are fundamental for advancing our understanding of the neurobiological adaptations elicited by sleep loss and the potential protective role of exercise.

The aim of this study was to investigate the metabolomic profiles of the cortex and hippocampus in mice under four conditions: control (C), sleep deprivation (SD), physical exercise (EX), and physical exercise followed by sleep deprivation (EX+SD), using ^1^H-NMR spectroscopy.

## Material and Methods

### Animals

The experimental protocol was carried out with a total of 48 adult male Swiss mice, weighing 25 to 30 g housed in standard conditions (12-h light/dark cycle, temperature 23±1°C, 60% humidity) with food and water *ad libitum*. The study followed the ethical principles of animal experimentation established by the National Council for the Control of Animal Experimentation (CONCEA) and was previously approved by the Ethics Committee of the Federal University of Ceara (No. 31/17). All experimental protocols were conducted in accordance with the guidelines established by the Ethical and Practical Principles for the Use of Laboratory Animals.

### Experimental design

The experimental design has been previously described (8). Animals were distributed into four groups (n=12, each). Group 1 (Control) was kept under normal conditions of accommodation in their cages. In Group 2 (EX), animals were exercised for 60 min/day, 5 days/week, on a motorized treadmill (EP 131 model - 8 lanes, Insight^®^ - Research and Education Equipment, Brazil) at 9 cm/s for 8 weeks. Group 3 (SD) was submitted to 72-h of SD using the modified multiple platform model. In Group 4 (EX+SD), animals were previously exercised (same protocol as Group 2) and immediately subjected to 72-h of SD (same protocol as Group 3). The EXE animals were given a 24-h rest period before the behavioral tests or the onset of SD (EXE+SD). Animals were euthanized, and the cerebral cortex and hippocampus were collected and immediately frozen in liquid nitrogen.

### Protocol of aerobic physical exercises on treadmill

The treadmill exercise training protocol was based on the study by Ferreira et al. (15). It started with a week of familiarization (5 min/day) to reduce adverse effects. The animals ran on a motorized treadmill with 8 separate tracks. This protocol consists of performing exercise 5 days a week with a progressive increase of 10 to 60 min over 2 weeks, at a speed of 9 cm/s.

### Sleep deprivation protocols

The SD protocol was based on the 72-h (72 h-SD) multiple platform method originally developed by Porsolt for rats and later adapted for mice (16). In this protocol, a group of 12 mice was placed in water tanks (41×34×16.5 cm) containing 14 small platforms (3 cm in diameter), with the water level maintained approximately 1 cm below the platform surface. Animals remained in this setup continuously for 72 h, preventing sustained sleep due to the risk of falling into the water upon muscle atonia. To mitigate non-specific stressors such as muscle fatigue or anxiety related to environmental exposure, animals underwent daily handling and brief habituation sessions on the platforms for three consecutive days prior to the onset of sleep deprivation. This acclimatization period allowed the animals to adapt to the restricted environment and ambient noise associated with the protocol, thereby reducing novelty-induced stress. Animals were able to move within the tank by jumping from one platform to another, thus avoiding social isolation. Food and water were provided *ad libitum* throughout the SD period.

### Animal euthanasia and brain tissue removal

Euthanasia was performed by beheading with a guillotine. The cortex and hippocampus were quickly dissected. Samples were immediately frozen in liquid nitrogen. All procedures were in accordance with CONCEA to minimize suffering and to use a minimal number of animals in the experiment.

### ^1^H-NMR analysis

Biological samples were prepared by combining the brain (cortex or hippocampus) of 4 mice to obtain one sample, resulting in a triplicate which represents the twelve mice per group. For the evaluation of the cortex, approximately 20 mg of the samples were used, while for the hippocampus, approximately 75 mg were used. The samples were solubilized in 600 μL D_2_O (99.9% deuterated water) containing 0.2 M Na_2_PO_4_/NaH_2_PO_4_ and 0.4 mM TMSP-2,2,3,3-d4 (sodium propionate-3-trimethylsilyl), used as chemical shift referencing (17). The ^1^H-NMR experiments were performed on the 600 MHz Agilent spectrometer^®^ equipped with a 5-mm inverse detection probe (HF/15N-31P, One Probe™, USA) with actively shielded Z gradient and by means of a simple single-pulse sequence for residual water saturation (Presat, Agilent terminology). Cortex and hippocampus data were obtained in triplicate on the same sample tube to confirm spectral consistency. For quantitative spectral acquisition, the probe was carefully tuned and matched, and the 90° hard pulse was calibrated at 299.15 K. Subsequently, an inversion-recovery pulse sequence was applied to estimate the longitudinal relaxation time (T1) of the sample nuclei. Based on the measured T1, the relaxation delay (D1) was set to 25 s and the acquisition time (AQ) to 5 s. Spectra were acquired using a receiver gain of 30 for the cortex and 56 for the hippocampus, with 64 transients averaged per sample, across a spectral width of 16 ppm and a total of 48 k data points. The spectra were processed using the Agilent VNMRJ^TM^ software by applying exponential multiplication of the free induction decays (FIDs) by a factor of 0.3 Hz and Fourier transformation of 16 k points. The phase correction was carried out manually, and the baseline correction was applied over the entire spectral range. The identification of the constituents was carried out using 2D NMR, such as gradient correlation spectroscopy (^1^H-^1^H COSY), single heteronuclear gradient quantum coherence (^1^H-^13^C HSQC), and gradient heteronuclear multiple bond correlation (^1^H-^13^C HMBC). Molecular structures, ^1^H and ^13^C chemical shifts, multiplicity, correlations and constant coupling, acquisition, and processing of 2D NMR data are available in the Supplementary Material, an open access database (www.hmdb.ca) (18), and literature reports (19). The complete signal assignments are shown in Supplementary Table S1.

### Chemometric analysis

The ^1^H-NMR spectra were converted to ASCII files (American Standard Code for Information Interchange) to create the matrices using the region from 0.34 to 9.11 ppm for both brain areas under analysis (cortex or hippocampus) and excluding the regions of water interference (from 4.65 to 4.90 ppm) and residual methanol (3.33 to 3.38 ppm). The matrices were imported individually into the PLS Toolbox™ program (version 8.6.2, Eigenvector Research Incorporated, USA) for chemometric analysis. For spectral pretreatments, algorithms for baseline correction such as normalization and alignment of variables using COW (Correlation Optimized Warping) with a segment of 20 data points and a gap of 10 data points were applied before the matrix decomposition (20). After pre-treatments, the sample data were centered on the mean, and the Singular Value Decomposition algorithm (SVD) was applied to decompose the matrices for principal component analysis (PCA) modeling. Relevant information from the brain neurochemistry data sets was obtained from the first two main components (CP) for all methods, with a 95% confidence level. Outliers were determined by visual inspection of Q residuals × Hotelling's T^2^ plot and replicates with both Hotelling T^2^ and Q residuals values higher than 1 were removed.

### Univariate statistical analysis

Organic compounds that showed relevance for the PCA distinction were quantified using an external reference method provided by the VnmrJ™ software (version 4.2, Agilent) (21). The combined uncertainties related to the concentration of malic acid, taurine, gamma-aminobutyric acid (GABA), choline, acetic acid, n-acetylaspartic acid, alanine, and lactic acid were estimated based on the analytical errors and the standard deviations from the sampling and acquisition replicates. In order to statistically certify the compounds' variability among the samples, the quantitative results were evaluated using one-way ANOVA, means were compared using the Tukey test, and the variance homogeneity among the groups was verified with the Levene test. A significance level of 0.05 was used. ANOVA analyses were performed using the Origin™ software version 9.4 (OriginLab Corporation, USA).

### Metabolic pathway analysis

To evaluate the effect of recurrent aerobic exercise and sleep deprivation in different parts of the brain of mice (cortex or hippocampus), the classification model using the discriminant analysis algorithm of orthogonal partial least squares (OPLS-DA) was applied using the PLS Toolbox^TM^ program (version 8.6.2, Eigenvector Research Incorporated). For paired comparison, the most distinct groups observed in the PCA were used, i.e., control *versus* aerobic exercise and sleep deprivation for the cortex and control *versus* sleep deprivation and sleep deprivation with aerobic exercise for the hippocampus. The loading graphs were analyzed and the variables important for the projection (VIP) with a value greater than 2 were considered (see Supplementary Figure S1). Therefore, the variables that presented importance for PCA, did not show any overlapped peaks, presented significant differences by ANOVA, and presented VIP greater than 2 were inputted for the analysis of impacted metabolic pathways using MetaboAnalyst 4.0 (https://www.metaboanalyst.ca/) (22). The *t*-test with false discovery rate (FDR) correction was used to determine significant changes between the groups. The Kyoto Encyclopedia of Genes and Genomes (KEGG™) database was used to analyze and visualize the most affected pathways. Normalization by sum and autoscaling was performed on the quantified compounds, and the model library for Mammals, *Mus musculus* (house mouse) was used.

## Results

**Figure 1:**
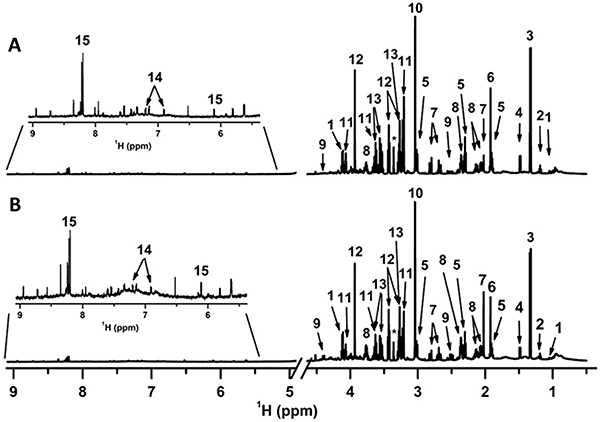
^1^H-NMR spectrum of (**A**) cortex and (**B**) hippocampus. 1: valine; 2: ethanol; 3: lactic acid; 4: alanine; 5: gamma-aminobutyric acid (GABA); 6: acetic acid; 7: n-acetyl-aspartic acid; 8: glutamate; 9: malic acid; 10: creatine; 11: choline; 12: taurine; 13: myo-inositol; 14: tyrosine; 15: inosine. *Residual methanol. Metabolite identification and NMR signal assignments were based on previously published ^1^H-NMR metabolomics studies and the HMDB database (1-5).

The cortex and hippocampus of the brain of mice submitted to recurrent aerobic exercise and sleep deprivation were analyzed. Figure 1A and B illustrate ^1^H-NMR spectra of the cortex and hippocampus, respectively, with a total of 15 identified compounds that included amino acids, short-chain organic acids, alcohols, and vitamins (see molecular structures, ^1^H and ^13^C chemical shifts, multiplicity and constant coupling in Supplementary Table S1).

**Figure 2:**
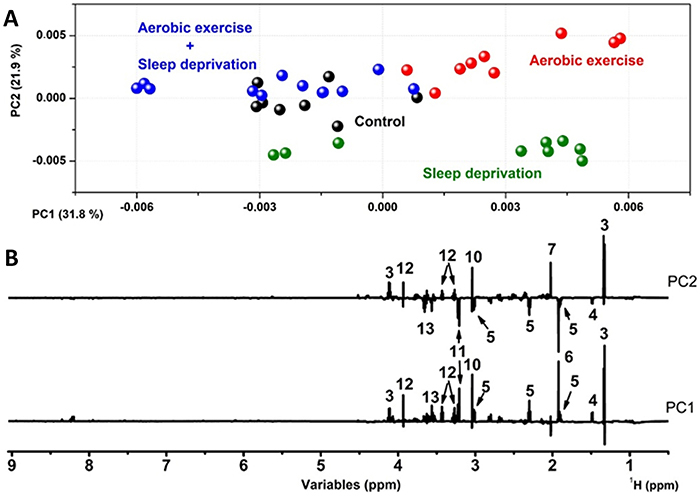
Principal component analysis (PCA) results from the ^1^H NMR dataset of the cortex: **A**, PC1×PC2 scores plot; **B**, respective loadings of PC1 and PC2 plotted in lines form with the identified compounds. 3: lactic acid; 4: alanine; 5: gamma-aminobutyric acid (GABA); 6: acetic acid; 7: n-acetyl-aspartic acid; 10: creatine; 11: choline; 12: taurine; 13: myo-inositol.

Due to the high dimensionality of the data set and the similarity inherent to the composition of the samples of each matrix, a chemometric evaluation was developed for a deep understanding of brain variability according to aerobic exercise and sleep deprivation. Therefore, PCA was applied to the numerical ^1^H-NMR matrix from cortex or hippocampus. Figure 2A illustrates the PCA scores of the cortex that retained 54.7% of the total variation in the first two PCs. Figure 2B illustrates the PC1 and PC2 loadings with the important metabolites for distinguishing the cortex samples according to the experimental design.

**Figure 3:**
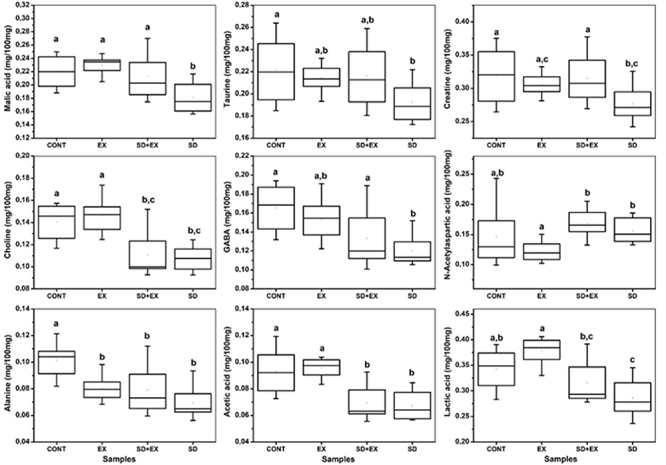
Boxplots representing the concentrations (mg/100 mg tissue) of malic acid, taurine, creatine, choline, γ-aminobutyric acid (GABA), N-acetylaspartic acid (NAA), alanine, acetic acid, and lactic acid in the cortex of four experimental groups: Control (CONT), Exercise (EX), Sleep Deprivation + Exercise (SD+EX), and Sleep Deprivation (SD). Data are reported as medians and interquartile range. Different letters above the boxplots indicate statistically significant differences among groups (P<0.05; Tukey test).

Figure 2A shows trends of separation between the cortex samples from the groups according to the PC1 and PC2 axis, with the cortex submitted to aerobic exercise tending to positive PC1 scores and the other samples with null PC1 values. The loads show that recurrent aerobic exercise induced increased levels of several metabolites including lactic acid, alanine, GABA, acetic acid, creatine, choline, taurine, and myo-inositol. The PC2 axis shows the cortex subjected to sleep deprivation with negative PC2 scores and the other samples with positive PC2 scores. The loadings' graph show that sleep deprivation induced an increase in alanine, GABA, choline, and myo-inositol and a decrease in lactic acid, n-acetylaspartic acid, creatine, and taurine in the cortex. The aforementioned molecules were quantified in order to attest the significant difference among groups. In addition, it is important to note that the cortex of the control samples (without exercise or sleep deprivation) showed a similar composition to those submitted to aerobic exercise followed by sleep deprivation at negative PC1 scores. Figure 3 illustrates the quantified compounds malic acid, taurine, creatine, choline, GABA, n-acetylaspartic acid, alanine, acetic acid, and lactic acid in the cortex.

**Figure 4:**
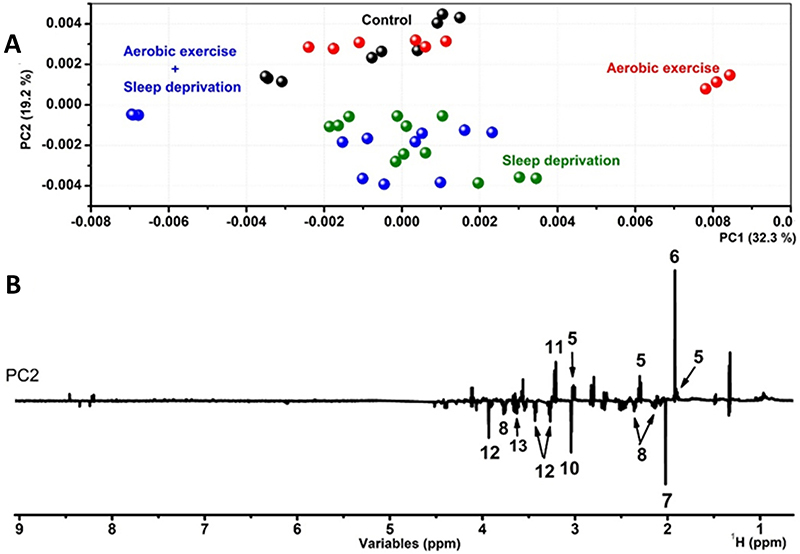
Principal component analysis (PCA) results from the ^1^H NMR dataset of the hippocampus: **A**, PC1×PC2 scores plot; **B**, respective loadings of PC2 plotted in lines form with the identified compounds. 5: γ-aminobutyric acid (GABA); 6: acetic acid; 7: N-acetylaspartic acid; 8: glutamate; 10: creatine; 11: choline; 12: taurine; 13: myo-inositol.

Figure 4 illustrates the multivariate results for hippocampus tissue of the groups, in which PCA retained 50.6% of the total variation in two first PCs. Figure 4A shows the score plot and Figure 4B illustrates the PC2 loading graphs with the important metabolites for the different samples. The hippocampus showed trends only on the PC2 axis, which showed a different response to aerobic exercise and SD.

**Figure 5:**
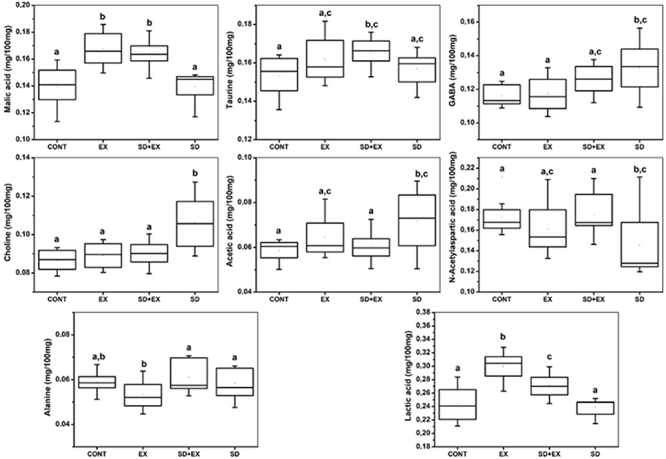
Boxplots representing the concentrations (mg/100 mg tissue) of malic acid, taurine, γ-aminobutyric acid (GABA), choline, acetic acid, N-acetylaspartic acid (NAA), alanine, and lactic acid in the hippocampus of four experimental groups: Control (CONT), Exercise (EX), Sleep Deprivation + Exercise (SD+EX), and Sleep Deprivation (SD). Data are reported as medians and interquartile range. Different letters above the boxplots indicate statistically significant differences among groups (P<0.05; Tukey test).

SD and aerobic exercise followed by SD had negative PC2 scores and the remaining samples had null and positive PC2 scores. PC2 loads showed that SD induced increased n-acetyl-aspartic acid, glutamate, creatine, taurine, and myo-inositol, while it decreased the levels of GABA, acetic acid, and choline (11) in the hippocampus. In addition, it is important to note that the hippocampus of the control samples (without exercise or sleep deprivation) had a similar composition to those submitted to aerobic exercise. The aforementioned molecules were quantified in order to attest the differences among the groups. Figure 5 shows the quantified compounds malic acid, taurine, GABA, choline, acetic acid, n-acetylaspartic acid, alanine, lactic acid in the hippocampus.

### Analysis of metabolic pathways

**Figure 6:**
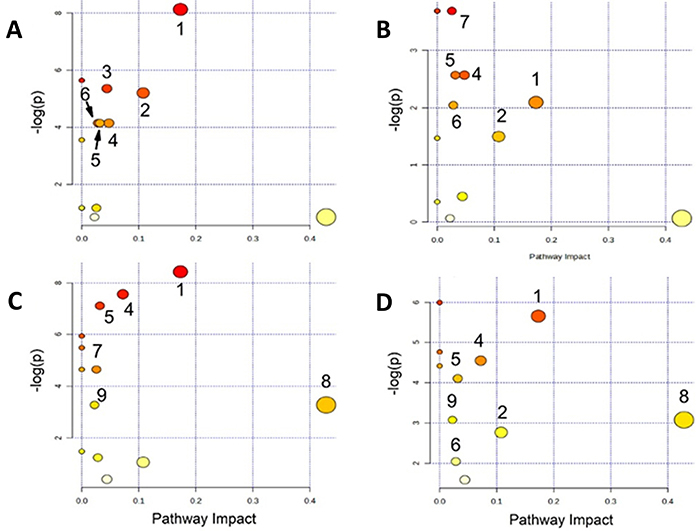
Pathways associated with the metabolism response to interventions in the brain. **A**, Cortex submitted to aerobic exercise; **B**, cortex subjected to sleep deprivation; **C**, hippocampus subjected to sleep deprivation; **D**, hippocampus subjected to aerobic exercise followed by sleep deprivation. 1: Alanine, aspartate, and glutamate metabolism; 2: Pyruvate metabolism; 3: Citrate cycle (TCA cycle); 4: Arginine and proline metabolism; 5: Butanoate metabolism; 6: Glycolysis or Gluconeogenesis; 7: Glycerophospholipid metabolism; 8: Taurine and hypotaurine metabolism; 9: Primary bile acid biosynthesis. The pathways are organized according to the P-value on the y-axis, reflecting the results of the pathway enrichment analysis and the pathway impact values on the x-axis, which represent the topology analysis. The color of each node corresponds to its P-value (with red indicating the lowest P-value and highest statistical significance), while the size of each node reflects the pathway impact factor, with larger circles indicating greater impact.

For a comprehensive analysis of the metabolic profile of the cerebral cortex and hippocampus of the different samples, supervised PLS was performed with peer comparison followed by OPLS-DA (23). Pathways with a false discovery rate (P<0.05) and with metabolites with impact on the pathway were considered as the main affected routes. In Figure 6, deep red circles indicate metabolites with an increased concentration and the size of the circle indicates the number of metabolites that were affected within that route. For more information of the parameters, see Supplementary Table S2. For paired comparison, the most distinct groups observed in the PCA were used, i.e., control *versus* exercise and SD for the cortex and control *versus* SD and aerobic exercise with SD for the hippocampus. Figure 6A and B shows the pathways associated with metabolic response in the cortex for control *versus* aerobic exercise and control *versus* SD. Figure 6C and D show the pathways in the hippocampus for control *versus* SD and control *versus* exercise followed by SD.

Comprehensively, the same metabolites were triggered by the interventions performed, regardless of the part of the brain under analysis. In the cortex, 6 metabolic pathways were triggered by aerobic exercise and SD, while in the hippocampus, 6 metabolic pathways were triggered by SD and 7 were triggered by SD after aerobic exercise.

## Discussion

Understanding brain functions and their associated pathologies has long been a central objective in neuroscience research, with various spectroscopic techniques playing a pivotal role in these investigations (24). Among these, metabolomics has emerged as a rapidly expanding field, garnering increasing attention for its ability to provide comprehensive insights into cellular metabolism and brain biochemistry (25). In this context, our study investigated brain metabolic alterations induced by SD, while evaluating the modulatory effects of prior physical exercise using ^1^H-NMR-based metabolomics. This approach enabled the identification of specific neurochemical shifts associated with SD and exercise, contributing valuable information to the understanding of their mechanistic interplay in the brain.

The overall metabolic composition of the cortex and hippocampus was qualitatively similar. The most abundant metabolites quantified were taurine, creatine, and lactic acid, consistent with previous studies (19). Through our experimental design, 15 metabolites were identified, nine of which exhibited significant alterations in response to the interventions. These included malic acid, taurine, choline, creatine, GABA, N-acetylaspartic acid, acetic acid, alanine, and lactic acid. Each of these compounds plays unique and critical roles in brain metabolism, neurotransmission, osmotic balance, or structural integrity.

In the cortex, recurrent aerobic exercise led to elevated levels of key metabolites such as lactic acid, alanine, GABA, acetic acid, creatine, choline, taurine, and myo-inositol. These changes reflect enhanced glycolytic activity, mitochondrial function, and neuroprotective adaptations driven by sustained physical training. Moreover, exercise preserved or partially restored metabolite levels that were diminished under SD, including malic acid, creatine, GABA, and choline, suggesting a stabilizing effect on cortical bioenergetics and synaptic signaling. These findings align with previous literature emphasizing exercise-induced mitochondrial efficiency and glial-neuronal metabolic coupling (26-28).

Sleep deprivation alone, however, triggered significant disruptions in cortical metabolism, characterized by decreased levels of lactic acid, malic acid, creatine, taurine, and choline, alongside increased concentrations of GABA, alanine, N-acetylaspartic acid, and myo-inositol. These alterations are indicative of impaired glycolysis, compromised oxidative metabolism, and compensatory glial responses. In particular, elevated GABA and myo-inositol levels may reflect increased inhibitory tone and astrogliosis in response to excitotoxic or inflammatory stimuli (29,30).

Interestingly, mice subjected to prior exercise followed by SD (EXE+SD) exhibited a cortical metabolic profile closely resembling the control group. This suggests that physical training conferred metabolic resilience, possibly by preserving levels of taurine, creatine, malic acid, and lactic acid, metabolites crucial for neuroenergetic homeostasis, osmotic regulation, and neurotransmitter synthesis. These data support the concept of exercise-induced neuroprotection through preconditioning mechanisms (31,32).

In the hippocampus, SD resulted in increased concentrations of N-acetylaspartate, glutamate, creatine, taurine, and myo-inositol, coupled with decreased levels of GABA, choline, and acetic acid. This profile suggests a shift toward excitatory signaling, osmolyte accumulation, and disrupted membrane metabolism. Such changes may underlie cognitive and emotional disturbances typically associated with sleep loss. In particular, the reduction of GABA and choline may impair hippocampal inhibitory control and acetylcholine-mediated plasticity (33).

Exercise alone promoted beneficial shifts in hippocampal metabolism, notably increasing taurine and maintaining malic acid levels. When administered prior to SD, exercise attenuated SD-induced metabolic disturbances, particularly in GABA, N-acetylaspartate, and acetic acid levels. These results underscore the role of exercise in enhancing hippocampal resilience to stress via improved energy regulation and neurochemical balance (34,35).

In addition to its well-known sleep-promoting effects, the adenosine A_1_ receptor plays a central role in regulating neuronal energy metabolism. *Ex vivo* studies using hippocampal slices demonstrated, via NMR spectroscopy, that A_1_ receptor activation reduces glucose consumption and lactate production during hypoxia, whereas its inhibition enhances extracellular lactate and glutamate levels (36). These findings highlight that A_1_-mediated signaling not only conserves energy via metabolic downregulation but also modulates the interplay between neurotransmission and astrocytic support. In our model, alterations in metabolites such as lactate, glutamate, and N-acetylaspartate in the cortex and hippocampus of SD-exposed mice may reflect increased endogenous adenosine levels and A_1_ receptor activation. This mechanism likely contributes to reduced glycolytic flux and shifts in substrate utilization, supporting the hypothesis that A_1_ receptor modulation integrates metabolic adaptation and synaptic regulation under sleep-deprived conditions.

From a pathway-level perspective, both SD and exercise modulated several overlapping but distinct metabolic circuits. In the cortex, SD activated pathways involved in alanine, aspartate, and glutamate metabolism; pyruvate metabolism; arginine and proline metabolism; butanoate metabolism; glycolysis/gluconeogenesis; and glycerophospholipid metabolism. Exercise primarily affected the TCA cycle, pyruvate metabolism, and glutamatergic pathways. In the hippocampus, SD impacted taurine and hypotaurine metabolism as well as primary bile acid biosynthesis, indicating region-specific metabolic stress responses. These insights reveal systemic neurochemical adaptations triggered by sleep loss and physical training (37,38).

NMR spectroscopy is a powerful analytical tool in metabolomics, providing high reproducibility, non-destructive measurements, and simultaneous detection of multiple metabolites with minimal sample preparation. However, traditional liquid-state NMR requires extraction procedures that can result in loss of labile compounds or alteration of the native biochemical environment. To overcome these limitations, high-resolution magic angle spinning (HR-MAS) NMR enables the direct analysis of intact tissue samples. HR-MAS minimizes line broadening caused by tissue heterogeneity, producing high-resolution spectra while preserving spatial and structural integrity. Thus, future applications of this protocol may benefit from adopting HR-MAS for more accurate tissue metabolite profiling.

We acknowledge that the 90-s interval between decapitation and tissue freezing may have permitted partial degradation of metabolites, as noted by Dienel (39). Nonetheless, all experimental groups were processed using an identical, standardized protocol, mitigating relative biases. Furthermore, comparisons with longitudinal *in vivo* data from aged mice (36) revealed consistent trends in metabolite variation despite marginally reduced absolute levels. Although acute stress from unanesthetized decapitation may influence specific metabolites (40), simultaneous handling of all groups likely ensured uniform exposure and minimized experimental variability.

## Conclusions

In summary, our ^1^H-NMR-based metabolomic analysis demonstrated that prior physical exercise effectively modulated neurochemical responses to sleep deprivation, particularly by preserving key metabolic intermediates in the cortex, such as lactate, creatine, and malic acid. These findings suggest that the neuroprotective effects of exercise are more pronounced in the cortex, while the hippocampus exhibits region-specific metabolic adaptations with partial responsiveness to exercise intervention.

Altogether, our results reinforce the role of regular physical activity as a modulator of brain energy metabolism and stress resilience. The partial attenuation of SD-induced metabolic disruption highlights the therapeutic potential of exercise in maintaining neurochemical homeostasis. Future studies should further explore the molecular mechanisms involved and evaluate the translational relevance of these findings in clinical and aging populations.

## Data Availability

All data generated or analyzed during this study are included in this published article (or in its supplementary information files).

## Supplementary Materials

Supplementary Material Click here to view [pdf].
