# Supplementary material for: 1H-NMR-based metabolomics reveals that prior exercise modulates metabolic changes in the cerebral cortex and hippocampus in sleep-deprived mice
Source: Braz J Med Biol Res. 2026 Mar 30;59:e14816. doi: 10.1590/1414-431X2025e14816 (PMC13037829; doi:10.1590/1414-431X2025e14816)
Supplement: Supplementary Material [file 1414-431X-bjmbr-59-e14816-suppl.pdf]

Figure S1. Variables important for the projection (VIP) loadings of the orthogonal partial least squares (OPLS-DA) for each pair wise comparison. VIP loadings for **A**, hippocampus control *vs* aerobic exercise followed by sleep deprivation; **B**, hippocampus control *vs* sleep deprivation; **C**, cortex control *vs* sleep deprivation; **D,** cortex control *vs* aerobic exercise.

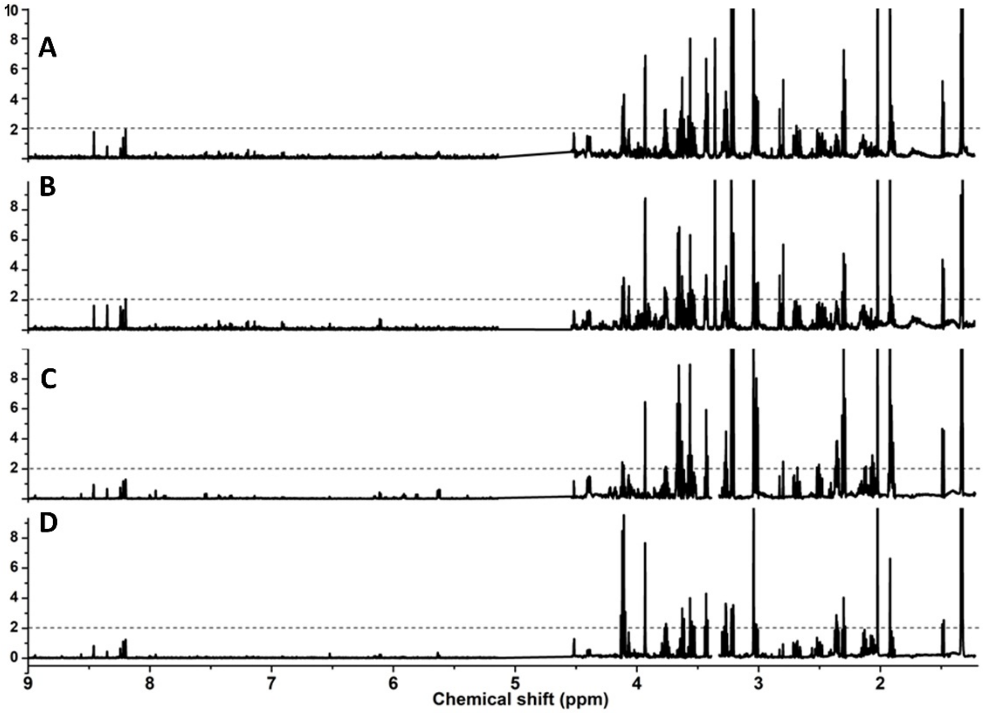

Table S1. Organic compounds identified in the brain of mice (cortex and hippocampus).

Structures	δ ^{1}H (multip.* J in Hz)	δ ^{13}C (HSQC)	δ^{1}H ref.	δ^{13}C ref.
**Amino acids**				
Alanine	2–3.80 (o)	53.5	3.90 (q 7.3)	53.4
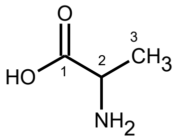	3–1.49 (d 7.2)	19.1	1.52 (d 7.3)	19.1
Valine	2–no	no	3.82 (d 4.4)	n
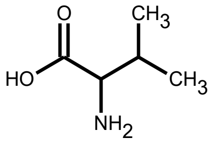	3–1.93 (o)	31.9	2.33 (m)	32.0
	4–1.00 (d 7.2)	19.5	1.02 (d 7.1)	19.1
	5–1.05 (d 7.2)	20.8	1.06 (d 7.1)	20.9
Glutamate	4–2.33 to 2.40 (m)	36.4	2.45 (m)	33.9
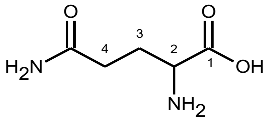	3–2.02 to 2.10 (m)	30.0	2.12 (m)	29.3
	3'–2.10 to 2.17 (m)	30.0		
	2–3.73 (o)	57.1	3.77 (o)	57.2
GABA	4–3.01 (t 7.2)	42.2	2.99 (t 7.6)	42.2
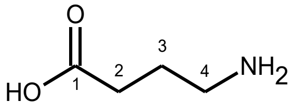	3–1.91 (o)	26.5	1.88 (quin 7.6)	26.3
	2–2.31(t 7.2)	37.2	2.28 (t 7.6)	37.1
Tyrosine	6,8–6.92 (m)	118.9	6.89 (m)	118.9
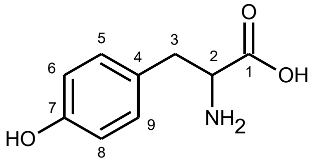	5,9–7.22 (m)	133.6	7.19 (m)	133.5
	2–(o)	no	3.93 (dd)	59.0
	3–no	no	3.06 (dd)	38.3
**Organic acids**				
Lactate	2–4.11 (o)	71.3	4.10 (q 6.93)	71.4
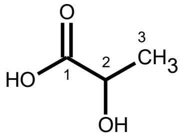	3–1.33 (d 7.20)	22.9	1.32 (d 6.93)	22.9
Malic	3–2.51 (d 15.6; 10.2)	42.5	2.34 (dd 15.4; 10.11)	45.5
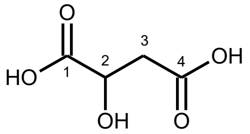	3'–2.77 (d 15.6; 3.8)		2.65 (dd 15.4; 2.9)	
	2–4.40(d 10.1; 3.8)	42.5	4.28 (d 10.1; 2.9)	45.5
		no		73.2
Acetic	2–1.92 (s)	26.9	1.90 (s)	26.1
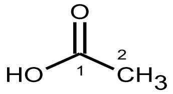				
**Other compounds**				
Ethanol	1–3.83 (o)	60.4	3.64 (o)	60.3
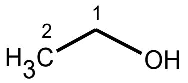	2–1.19 (t, 7.2)	19.6	1.17 (o)	19.6
Creatine	2–3.05 (s)	39.9	3.03 (s)	39.5
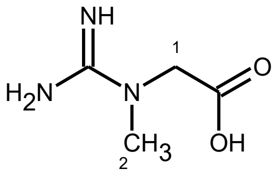	1–3.92 (o)	56.9	3.92 (s)	56.4

Taurine 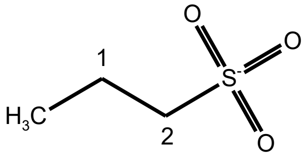	2–3.27 (t 6.6)<br>1–3.44 (t 6.6)	50.4<br>38.3	3.24 (t 6.6)<br>3.40 (t 6.6)	50.4<br>38.3
N-acetylaspartic acid (NAA) 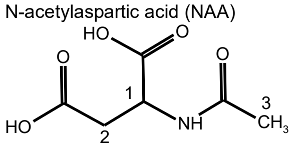	1–4.37 (o)<br>2–2.59 (d, 16.8)<br>2'–2.70 (d, 16.8)<br>3–2.02 (s)	55.7<br>40.5<br>40.5<br>25.0	1–4.37 (m)<br>2–2.59 (dd, 15.7; 9.9)<br>2'- 2.70 (dd, 15.7; 3.7)<br>3–2.02 (s)	56.2<br>42.4<br><br>42.4<br><br>24.7
Myo-inositol 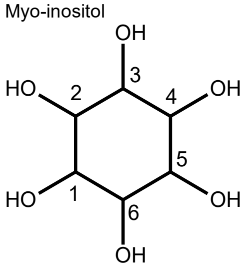	1–4.07 (t)<br>2–3.61 (t 9.6)<br>3–3.52 (dd 9.6; 3.1)<br>4–3.27 (o)	75.2<br>75.5<br><br>74.1<br>77.3	1–4.05 (t 9.7)<br>2–3.61 (t 9.7)<br>3–3.52 (dd 9.7; 2.8)<br>4–3.27 (t 2.8)	74.9<br>75.1<br><br>73.9<br>77.1
Choline 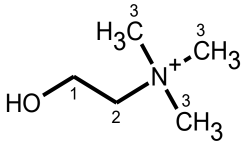	3–3.22 (s)<br>2–3.60 (o)<br>1–3.91 (o)	57.0<br>69.3<br>57.9	3.19 (s)<br>3.50 (dd 5.8; 4.2)<br>4.05 (m)	56.7<br>70.1<br>58.5
Inosine 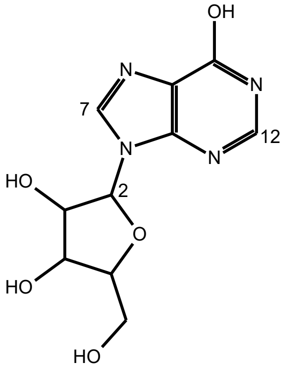	2–6.11 (d 5.8)<br>7–8.22 (s)<br>12–8.20 (s)	no<br>no<br>no	2–6.05 (d 5.7)<br>7–8.31 (s)<br>12–8.19 (s)	90.9<br>142.7<br>148.9

s: simplet; d: duplet; t: triplet; q: quadruplet; quin: quintet; dd: double of duplets; dt: double of triplets; o: overlapping signal; n: no information; no: not observed. ^{1}H and ^{13}C chemical shift assignments were confirmed by comparison with previously reported data in Graham et al. (1), Liu et al. (2), Quansah et al. (3), Wishart et al. (4), and Wesley et al. (5).

Table S2. Parameters from the pathway analysis.

Pathway name	Match status	P	–log(p)	Holm p	FDR	Impact
Cortex – Control × Exercise						
Alanine, aspartate, and glutamate metabolism	3/28	7.3818E-9	8.1318	9.5964E-8	9.5964E-8	0.17308
Citrate cycle (TCA cycle)	1/20	4.3471E-6	5.3618	4.7818E-5	1.8837E-5	0.04412
Pyruvate metabolism	3/23	6.1789E-6	5.2091	6.1789E-5	2.0082E-5	0.10781
Glycolysis or Gluconeogenesis	2/26	7.0369E-5	4.1526	6.3332E-4	1.3101E-4	0.02831
Arginine and proline metabolism	1/36	7.0542E-5	4.1516	6.3332E-4	1.3101E-4	0.04767
Butanoate metabolism	1/15	7.0542E-5	4.1516	6.3332E-4	1.3101E-4	0.03175
Cortex – Control × Sleep-deprived						
Glycerophospholipid metabolism	1/36	2.0289E-4	3.6927	0.0026376	8.792E-4	0.02569
Arginine and proline metabolism	1/36	0.002694	2.5696	0.02694	0.0070044	0.04767
Butanoate metabolism	1/15	0.002694	2.5696	0.02694	0.0070044	0.03175
Alanine, aspartate, and glutamate metabolism	3/28	0.008074	2.0929	0.064592	0.01686	0.17308
Glycolysis or Gluconeogenesis	2/26	0.0090782	2.042	0.064592	0.01686	0.02831
Pyruvate metabolism	3/23	0.032078	1.4938	0.19247	0.049467	0.10781
Hippocampus – Control × Sleep-deprived						
Alanine, aspartate, and glutamate metabolism	3/28	3.7922E-9	8.4211	4.9298E-8	4.9298E-8	0.17308
Arginine and proline metabolism	2/36	2.7999E-8	7.5529	3.3598E-7	1.8199E-7	0.07209
Butanoate metabolism	1/15	7.837E-8	7.1058	8.6207E-7	3.396E-7	0.03175
Glycerophospholipid metabolism	1/36	2.2827E-5	4.6415	1.8262E-4	4.2394E-5	0.02569
Taurine and hypotaurine metabolism	1/8	5.3757E-4	3.2696	0.0032254	7.765E-4	0.42857
Primary bile acid biosynthesis	1/46	5.3757E-4	3.2696	0.0032254	7.765E-4	0.02239
Hippocampus – Control × Sleep-deprived plus exercise						
Glycerophospholipid metabolism	1/36	1.7944E-7	6.7461	2.3327E-6	1.1663E-6	0.02569
Alanine, aspartate, and glutamate metabolism	3/28	2.2141E-6	5.6548	2.2141E-5	7.196E-6	0.17308
Arginine and proline metabolism	2/36	2.8297E-5	4.5483	2.2638E-4	6.131E-5	0.07209
Butanoate metabolism	1/15	7.9005E-5	4.1023	4.7403E-4	1.2838E-4	0.03175
Taurine and hypotaurine metabolism	1/8	8.4493E-4	3.0732	0.0042247	0.0010984	0.42857
Primary bile acid biosynthesis	1/46	8.4493E-4	3.0732	0.0042247	0.0010984	0.02239
Pyruvate metabolism	3/23	0.001729	2.7622	0.005187	0.0020433	0.10781
Glycolysis or Gluconeogenesis	2/26	0.0091383	2.0391	0.018277	0.0098998	0.02831
Citrate cycle (TCA cycle)	1/20	0.026173	1.5821	0.026173	0.026173	0.04412

Selection and interpretation of metabolic pathways were supported by previously published ^{1}H-NMR brain metabolomics studies (1–3,5).

References

1. Graham SF, Holscher C, Green BD. Metabolic signatures of human Alzheimer's disease (AD): ^{1}H NMR analysis of the polar metabolome of post-mortem brain tissue. Metabolomics. 2014;10:744-53, doi: 10.1007/s11306-013-0610-1.
2. Liu J, Sheldon RA, Segal MR, Kelly MJS, Pelton JG, Ferriero DM, et al. ^{1}H nuclear magnetic resonance brain metabolomics in neonatal mice after hypoxia–ischemia distinguished normothermic recovery from mild hypothermia recoveries. Pediatr Res. 2013;74(2):170-9, doi: 10.1038/pr.2013.88.
3. Quansah E, Ruiz-Rodado V, Grootveld M, Probert F, Zetterström TS. ^{1}H NMR-based metabolomics reveals neurochemical alterations in the brain of adolescent rats following acute methylphenidate administration. Neurochem Int. 2017;108:109-20, doi: 10.1016/j.neuint.2017.03.003.
4. Wishart DS, Jewison T, Guo AC, Wilson M, Knox C, Liu Y, et al. HMDB 3.0—the human metabolome database in 2013. Nucleic Acids Res. 2012;41:D801-D807, doi: 10.1093/nar/gks1065.
5. Wesley UV, Bhute VJ, Hatcher JF, Palecek SP, Dempsey RJ. Local and systemic metabolic alterations in brain, plasma, and liver of rats in response to aging and ischemic stroke, as detected by nuclear magnetic resonance (NMR) spectroscopy. Neurochem Int. 2019;127:113-24, doi: 10.1016/j.neuint.2019.01.025.
